# Heterophilic antibodies in sera from individuals without loxoscelism cross-react with phospholipase D from the venom of *Loxosceles* and *Sicarius* spiders

**DOI:** 10.1186/s40409-018-0155-x

**Published:** 2018-07-26

**Authors:** Tomás Arán-Sekul, José M. Rojas, Mario Subiabre, Victoria Cruz, William Cortés, Luis Osorio, Jorge González, Jorge E. Araya, Alejandro Catalán

**Affiliations:** 1 Laboratory of Molecular Parasitology, Department of Medical Technology, Faculty of Health Sciences, University of Antofagasta, 1270300 Antofagasta, Chile; 2 Cellular and Molecular Physiology Laboratory (CMPL), Division of Obstetrics and Gynecology, School of Medicine, Faculty of Medicine, Pontifical Catholic University of Chile, 8330024 Santiago, Chile

**Keywords:** *Loxosceles laeta*, Heterophilic antibodies, Natural antibodies, *Sicarius*

## Abstract

**Background:**

Loxoscelism is a severe human envenomation caused by *Loxosceles* spider venom. To the best of our knowledge, no study has evaluated the presence of antibodies against *Loxosceles* venom in loxoscelism patients without treatment with antivenom immunotherapy. We perform a comparative analysis for the presence of antibodies capable of recognizing *Loxosceles* venom in a group of patients diagnosed with loxoscelism and in a group of people without loxoscelism.

**Methods:**

The detection of *L. laeta* venom, *Sicarius* venom and recombinant phospholipases D from *Loxosceles* (PLDs) in sera from people with loxoscelism (Group 1) and from healthy people with no history of loxoscelism (Group 2) was evaluated using immuno-dot blot, indirect ELISA, and Western blot.

**Results:**

We found naturally heterophilic antibodies (IgG-type) in people without contact with *Loxosceles* spiders or any clinical history of loxoscelism. Either serum pools or single sera from Group 1 and Group 2 analyzed by dot blot tested positive for *L. laeta* venom. Indirect ELISA for venom recognition showed titles of 1:320 for Group 1 sera and 1:160 for Group 2 sera. Total IgG quantification showed no difference in sera from both groups. Pooled sera and purified IgG from sera of both groups revealed venom proteins between 25 and 32 kDa and the recombinant phospholipase D isoform 1 (rLlPLD1), specifically. Moreover, heterophile antibodies cross-react with PLDs from other *Loxosceles* species and the venom of *Sicarius* spider.

**Conclusions:**

People without contact with the spider venom produced heterophilic antibodies capable of generating a cross-reaction against the venom of *L. laeta* and *Sicarius* spiders. Their presence and possible interference should be considered in the development of immunoassays for *Loxosceles* venom detection.

**Electronic supplementary material:**

The online version of this article (10.1186/s40409-018-0155-x) contains supplementary material, which is available to authorized users.

## Background

Loxoscelism is a clinical picture of poisoning produced by *Loxosceles* spiders venom that has a considerable impact on the population living in the Americas, from the United States to Chile [1, 2]. In countries such as Brazil, it is a serious public health problem, with a high number of cases reported annually, with some of them corresponding to fatal cases [3–5]. It is also considered a public health problem in Chile, where the most recent data from the Center for Toxicological Information at the Pontificia Universidad Católica de Chile (CITUC), showed that of 2831 possible cases in a year roughly 10% were confirmed as loxoscelism [6]. Loxoscelism is provoked by bites of spiders from the genus *Loxosceles* and its clinical presentation can progress from the local and most frequently dermal necrosis lesion, called cutaneous loxoscelism (CL), to a systemic condition less frequent, but more severe, called systemic or viscero-cutaneous loxoscelism (VCL). The clinical manifestations of loxoscelism (CL or VCL) depend on different factors, such as the amount and concentration of inoculated venom, the anatomical location of the bite, the susceptibility of the host, and the species and gender of the spider [7–9].

The diagnosis of loxoscelism is usually clinical and presumptive. This often depends on capture of the arachnid by the patient for later taxonomic identification in the laboratory, which helps guide an effective diagnosis [10]. However, this is rare, since patients do not bring the arachnid with them, making diagnoses dependent upon histological findings, epidemiology, signs and symptoms [11, 12]. In addition, there are no commercial diagnostic tests available, so the diagnosis is made according to the evolution of clinical symptoms [11, 13]. In this regard, the designing of these diagnostic assays may involve the careful attention in specificity, sensitivity, dynamic range, reproducibility and accuracy, but also require the identification of factors that may interfere with the assay leading to erroneous results [14].

Few efforts have been made in the development of a specific detection method for *Loxosceles* venom, which include a sandwich ELISA test for detection of *L. intermedia* venom on mice inoculated with *L. intermedia* venom and distinguish them from those inoculated with venom from *L. gaucho, L. laeta, P. nigreventer*, scorpions, and snakes. The test was able to detect 0.8 ng of venom per assay and could detect *L. intermedia* antigens in clinical serum samples from loxoscelism patients [15]. A second sandwich ELISA was developed for detecting *L. reclusa* venom using polyclonal rabbit antibodies, and could detect 0.1 ng of *Loxosceles* venom. However, cross-reactivity was detected with venom from other arachnid species not related to *Loxosceles* genus [16]. Despite this, its clinical efficacy was documented using a noninvasive sample from the lesions of loxoscelism patients [17, 18]. More recently, immunoconjugates of LimAb7 monoclonal antibody – specific against toxic components of *L. intermedia* venom – were evaluated for developing a competitive ELISA and sandwich ELISA for detecting *L. intermedia* venom. This assay had a detection limit of 39 ng/mL, however, was not able to detect venom from other species of *Loxosceles* as *L. laeta* or *L. gaucho* venom [19].

Although the specificity and sensitivity of immunoassays are important aspects to be considered and overcome in order to implement a diagnostic test for loxoscelism, other aspects such as the type of sample to be used and the presence of interfering factors should also be considered. Therefore, endogenous agents present in serum samples may interfere and cause false positive or false negative results. Important interfering agents in immunoassays are the endogenous antibodies, including: heterophilic antibodies (HA), human anti-animal antibodies (HAAA), and autoantibodies (AA). Heterophilic antibodies are naturally occurring antibodies present in individuals with no known exposure to specific antigens [20, 21]. These antibodies have low affinity and broad specificity, and their immunoassay interference mechanism occurs generally by cross-linking with captured antibodies or detection antibodies [22].

Up to this moment, only one study evaluated the presence of antibodies capable of recognizing *Loxosceles* venom present in loxoscelism patients who received antivenom therapy [23]. However, a detailed analysis of the presence of antibodies anti-*Loxosceles* venom has not been performed in patients diagnosed with loxoscelism and without antivenom therapy in order to rule out the participation in the venom detection of antibodies introduced in the antivenom therapy. Also, there has not been performed an evaluation of the possible presence of antibodies with cross-reaction potential for *Loxosceles* venom, or the presence of antibodies capable of interfering in specific immunoassays in people without loxoscelism.

In this study we performed a comparative analysis of the presence of antibodies capable of recognizing *Loxosceles* venom in a group of patients diagnosed with loxoscelism and in a group of people without contact with the spider or clinical history of loxoscelism to determine the relevance of using serum samples in the development of a rapid inmunotest for detection of *Loxosceles* venom. We found the presence of heterophilic antibodies capable of detecting the venom of *Loxosceles* and *Sicarius* spiders in both groups and discussed its origins and importance as a potential interference in diagnostic immunoassays for loxoscelism.

## Methods

### Serum sample collection from people with or without a clinical history of loxoscelism

Ten serum samples from people with a clinical history of loxoscelism and 30 serum samples from people without clinical history of loxoscelism or other arachnid-bite symptoms were collected during the period from March to December 2012, in the city of Antofagasta, Chile. Ten milliliters of peripheral blood were collected by venipuncture (informed consent was obtained from donors). Serum was collected through centrifugation at 2000×*g* for 10 min at 4 °C, and stored in aliquots of 500 μL at − 80 °C until use. Protocols for sample collection and informed consent were approved by the Ethics Committee in Scientific Research of the University of Antofagasta (CEIC-UA).

For the purpose of the study, samples from people with a clinical history of loxoscelism were part of the loxoscelism study group (Group 1), and samples from people without clinical history of loxoscelism were part of the control group (Group 2).

Group 1 was defined based on:

- loxoscelism diagnosis according to a clinical history;
- a patient’s own assertion of having been bitten by some arachnid (association with biting by *L. laeta*) with and without dermonecrotic or visceral loxoscelism;
- visual assessment of current or previous dermonecrotic lesions. People who reported being bitten by an arachnid were identified and confirmed for loxoscelism dermonecrotic lesions at the Molecular Parasitology Laboratory of the Faculty of Health Sciences of the Universidad de Antofagasta, according to the clinical guide for handling the bites of the corner spider from the Chilean Ministry of Health [24].

People in Group 2 were defined based on:

- no verifiable clinical history of loxoscelism or having previously suffered a bite from *L. laeta* or other type of arachnid;
- no presentation of chronic diseases or allergies;
- no evidence of autoimmune diseases, rheumatoid arthritis, or any known physical illness affecting their immunological status;
- no acute infectious processes at the time of sample extraction; and
- no presence of skin lesions attributable to infectious bacterial processes.

Among the samples in Group 1, 60% were from patients with cutaneous loxoscelism and 40% were from patients with viscero-cutaneous loxoscelism. The age range of people of groups 1 and 2 was 18 to 60 years old, and both groups had equal proportions of men and women (Table 1).

**Table 1:** Distribution of serum samples from patients groups with and without loxoscelism

Age group	Without loxoscelism^a^ (*n* = 30)		With loxoscelism^a^			
			Cutaneous^b^ (*n* = 6)		Systemic^b^ (*n* = 4)	
	Male	Female	Male	Female	Male	Female
18–29	11/36.7%	16/53.3%	1/16.7%	2/33.3%	2/50%	1/25%
30–60	3/10%	–	2/33.3%	1/16.7%	–	1/25%

^a^Samples of serum obtained under informed consent (procedure approved by the Research Ethics Committee of the Universidad de Antofagasta – CEIC-UA)

^b^According to clinical history and/or direct observation of dermonecrotic lesion. Samples are represented as number and percentage (n/%)

### Spider venom, recombinant phospholipase D expression and purification

The recombinant protein rLlPLD1 was expressed and purified as previously described [25]. In addition, the nucleotide sequences for different phospholipase D isoforms of the four representative species of *Loxosceles* (*L. laeta*, *L. intermedia*, *L. reclusa* and *L. gaucho*) available in GeneBank (NCBI) were used for expression of their ORFs in *E. coli* BL21 DE3 and purified as fusion proteins with a 6His tag at the N-terminus (LrSMD1 and LgDerProt1) or at the C-terminus (rLlPLD2 and LiDerTox1) by GenScript (GenScript Inc., USA). GenBank accession numbers for nucleotide sequences used and the molecular masses of the respective recombinant proteins were: *L. laeta* PLD isoform 2 (LlPLD2), access n° GU121906 [25], 32,055 Da with C-His tag; *L. reclusa* sphingomyelinase D isoform 1 (LrSMD1), access n° AY559846.1 [26], 31,219 Da with N-His tag; *L. intermedia* sphingomyelinase P1 (LiSMD P1), access n° AY304471.2 [27], 34,982 Da and C-His tag; and *L. gaucho* dermonecrotic protein 1 (LgDerProt1), access n° AY974250.1, 31,172 Da with N-His tag. Moreover, venom from 20 female *L. laeta* and 20 *Sicarius* adults was extracted by electrostimulation and collected as previously reported [28]. Polyclonal mouse anti-*L. laeta* venom antibodies were prepared as previously documented [25].

### Dot blot to determine *L. laeta* antivenom antibodies

Dot blot for *L. laeta* venom antibody detection was assembled in our laboratory, with 1 μg of *L. laeta* venom being adsorbed onto a nitrocellulose membrane using a 96-well Dot-Blot Filtration Manifold System (Gibco BRL). The presence of adsorbed proteins on the membrane was evaluated by staining with Ponceau red. The membrane was then blocked for 1 h at 22–25 °C with 5% non-fat milk in PBS/0.1% Tween20 (PBS-T). Each dot was then incubated with a pool of Group 1 or Group 2 sera (1:1000 dilution), and alternatively with single serums of Group 1 or Group 2 at a 1:10 dilution. The membranes were washed three times with PBS-T and incubated for 1 h at 22–25 °C with the anti-human IgG-HRP secondary antibody in 1:50,000 dilution, then washed again three times with PBS-T and developed by ECL.

PBS or pre-immune mouse serum (1:1000 dilution) was used as the negative control. As positive control, mouse anti-*L. laeta* venom serum (1:10,000 dilution) and monoclonal antibody 7E4-D2 anti-rL1PLD1 (1:50,000 dilution) were used [25]. BSA was used as unrelated antigen to evaluate specificity of reaction. Images were captured on a ChemiBis 2.0 DNR photo-documenter (DNR Bio-Imaging Systems Ltd., Jerusalem, Israel). Intensity of dots was evaluated by densitometry and expressed as relative density percentage.

### Indirect ELISA for detection of *L. laeta* venom antibodies

The titration of different sera was carried out using an indirect ELISA for detecting specific circulating antibodies against *L. laeta* venom, based on previously published protocols [29], and mounted in our laboratory. On 96-well ELISA microtiter plates (Nunc MaxiSorp™, Thermo Fisher Scientific), 1 μg of *L. laeta* venom in 0.02 M sodium carbonate/bicarbonate buffer (pH 9.6) per well was adsorbed, incubated at 37 °C for 1 h, and then at 4 °C for the overnight. Wells with *L. laeta* venom were blocked with 5% non-fat milk in PBS/0.05% Tween20 (PBS-T) for 1 h. The titer of sera from Group 1 and Group 2 was determined by using a two-fold serial dilution of serum samples from 1:10 to 1:5120 and incubated for 1 h at 37 °C.

Column 11 of the microplate was incubated with only PBS-T and used as a blank, while column 12 was used as specificity control with BSA adsorbed to the well. Subsequently, each well was washed four times with PBS-T and incubated for 1 h at 37 °C with anti-human IgG bound to peroxidase at 1:50,000 dilution (Sigma Aldrich Co, USA). After four washes with PBS-T and two washes with only PBS, 100 μL of the tetramethylbenzidine (TMB) substrate were added and incubated for 30 min at room temperature in the dark. The reaction was stopped by the addition of 100 μL of 3 N sulfuric acid (stop solution) and the absorbance of each well was measured at 450 nm in a BioRad model 550 microplate reader (BioRad, Hercules, USA). The background cut-off point was determined by the mean value of the blank absorbance (PBS-T) for at least 30 negative control replicates, plus three standard deviations (0.055 + 0.0088 = 0.0814). Reaction titers were determined as the inverse of the last positive reaction at the cut off value for each serum dilution. Samples from both groups showed normal distributions, according to the D’Agostino & Pearson omnibus normality test.

For IgG avidity ELISA, 100 μL of pooled serum of group 1 or 2 diluted 1:100 in buffer was added to each well coated with *L. laeta* venom in triplicate. After incubation for 1 h at 37 °C, wells were incubated with 6 M urea solution or PBS for 10 min. After four washes, wells were incubated with peroxidase labeled anti-human IgG for 1 h at 37 °C, then substrate was added, and the reactions were stopped after 30 min by addition of 100 μL of stop solution per well. Reactions were read at 450 nm, and avidity index was calculated by dividing absorbance of the wells treated with urea by that of untreated wells.

### IgG quantification in serum samples

Serum sample IgG antibody quantification was performed using radial immunodiffusion (RID) quantification, using the Diffu-Plate® kit for total IgG (Biocientifica S.A, Buenos Aires, Argentina), following the manufacturer’s instructions. For this, 5 μL of serum from each individual was seeded into each well of the immunodiffusion plate and incubated at room temperature (22–25 °C) for 48 h. The measurement of each immunoprecipitation halo was performed using a ruler with accuracy of 0.01 mm, and the total IgG concentration was determined by comparison against data provided by the manufacturer (batch 1157, plate range: 201.8–3645.7 mg/dL; adult reference value: 710–1520 mg/dL).

### Purification of IgGs and immunoadsorption of antibodies against *L. laeta* venom

Purification of IgG antibodies from sera was performed using the Pierce™ Protein G Agarose kit (Thermo Fisher Scientific, Inc. Waltham, MA, USA), following manufacturer’s instructions. Protein G Agarose resin in a 3:1 ratio with binding buffer (0.1 M sodium acetate, pH 5.0) was incubated with serum pools from groups 1 and 2, both previously diluted 1:1 in binding buffer and subsequently incubated in an orbital shaker at room temperature for 1 h and centrifuged at 500×*g* for 1 min. Then, each purification was washed twice with two volumes of PBS and again centrifuged at 500×*g* for 1 min, and the supernatant was discarded.

Finally, 1 mL of the elution buffer (0.1 M glycine-HCl buffer, pH 2.8) was added twice and incubated for 10 min at room temperature, with gentle mixing. The purification was neutralized with 50 μL of 1 M Tris, pH 8.0, per mL of obtained eluate, then concentrated in a Microcon® centrifugal filter (Merck-Millipore, Burlington, MA, USA) with a cut off of 10 kDa, and the elution buffer was exchanged for PBS (pH 7.3). The purified IgG antibodies were stored at − 20 °C. The purified IgG antibodies were evaluated by SDS-PAGE in gel at 10% and measuring absorbance at 280 nm in a TECAN® Infinite M200® PRO spectrofluorometer (Tecan Group Ltd., Männedorf, Switzerland). Subsequently, purified IgG antibodies were immunoselected using 2 μg of *L. laeta* venom adsorbed onto a nitrocellulose membrane in a 96-well Dot Blot Filtration Manifold system (Gibco BRL).

The presence of the adsorbed proteins to the membrane was evaluated by staining with Ponceau red. The membrane was then blocked with 5% non-fat milk in PBS-T for 1 h at 22–25 °C. Subsequently the membrane was washed with PBS-T and incubated with 10 mL (1:10 diluted) of purified IgG from groups 1 or 2 in PBS for 2 h at 4 °C. Next, IgGs that did not recognize *L. laeta* venom were washed three times in Borate-Saline wash buffer (0.1 M boric acid, 0.25 mM sodium tetraborate, 0.5 M NaCl, 0.05% Tween-20, pH 8.0) for 10 min with gentle mixing. IgG antibodies immunoselected against *L. laeta* venom were eluted during incubation with elution buffer (0.1 M glycine, 0.15 M NaCl, pH 2.6) for 5 min. Immediately, the IgG antibodies elution was neutralized with 50 μL of 1 M Tris-HCl, pH 8.0. Antibodies were concentrated using an Amicon® Ultra-15 centrifugal filter (Merck-Millipore, Burlington, MA, USA) with a cut-off of 10 kDa and the elution buffer was exchanged for PBS (pH 7.3). The *L. laeta* venom immunoselected IgG antibodies were stored at − 20 °C.

### Two-dimensional electrophoresis

Two-dimensional (2D) electrophoresis was performed using 100 μg of electrostimulated venom from *L. laeta*, which was first precipitated and resuspended in C1 buffer (8 M Urea, 1 M Thiourea, 4% CHAPS, 66 mM DTT, 0.5% ampholytes, pH range 3–10 NL). IPG strips (7 cm, pH 3–10 NL, Bio-Rad, Hercules, CA, USA) were rehydrated with samples in C1 buffer for 12 h at 20 °C. Isoelectric focusing was performed in the PROTEAN IEF Cell (Bio-Rad, Hercules, CA, USA) system until a total of 11,000 Vh^− 1^ was reached. After the first dimension was run, the strips were stored at − 80 °C until used. For the second dimension, the IPG strips were thawed at room temperature, then the proteins were subjected to a reduction treatment by incubating for 15 min in equilibrium buffer (50 mM Tris-HCl, pH 8.8, 6 M urea, 2% SDS, and 30% glycerol) containing DTT and then alkylated by incubating for 15 min in equilibrium buffer with iodoacetamide.

Finally, IPG strips were placed in 12% SDS-PAGE gels. Gels were stained with Coomassie Brilliant Blue G-250. To perform Western blot on the 2D electrophoresis-separated venom, 20 μg of venom was used and detected using 1 μg/mL of IgG antibodies purified from sera of Groups 1 and 2, washed, and then incubated with goat anti-human HRP-IgG antibody (1:50,000 dilution) in TBS-T for 1 h at room temperature, and the membranes were developed using the ECL™ Western blotting detection reagent kit (GE Healthcare, Chicago, IL, USA).

### Immunoblotting

Immunoblotting was performed by separating 5 μg of the different recombinant proteins (rLlPLD1 and rLlPLD2 from *L. laeta*, LiSMDP1 from *L. intermedia*, LrSMD1 from *L. reclusa,* and LgDerProt1 from *L. gaucho*), or 5 μg of *L. laeta* and *Sicarius* venom, using a 12% SDS-PAGE gels under non-reducing conditions. Additionally, 5 μg of phospholipase A_2_ (PLA_2_) from bee venom (*Apis mellifera*) (Sigma-Aldrich Co, St Louis, MO, USA) and phospholipase C (PLC) from *Bacillus cereus* (Sigma-Aldrich, USA) were tested. Gels were stained with Coomassie Brilliant Blue or transferred to a nitrocellulose membrane. After transfer, the membranes were blocked for 2 h with 5% non-fat milk in TBS/0.1% Tween20 (TBS-T) and incubated for 1 h at room temperature with pooled sera from Groups 1 and 2 (1:1000 dilution) or with purified and immunoselected IgGs from both groups at 1 μg/mL. Membranes were washed six times for 10 min each with TBS-T and incubated with goat anti-human HRP-IgG antibody (1:50,000 dilution) in TBS-T for 1 h at room temperature. After another six washes with TBS-T, the membranes were developed with the ECL™ Western blotting detection reagent kit (GE Healthcare, Chicago, IL, USA).

### Immunoprecipitation

Immunoprecipitation of *L. laeta* venom was done using Pierce™ Protein G Agarose (Thermo Fisher Scientific, Inc. Waltham, MA, USA), according the manufacturer’s instructions. For this, 100 μg of pure *L. laeta* venom was incubated with 5 μL (1:20 dilution) of pooled serum from Group 1 or Group 2 in 100 μL of IP buffer (25 mM Tris, 150 mM NaCl, pH 7.2) overnight at 4 °C. Another 100 μg of venom was incubated with 5 μL of mouse anti-*L. laeta* venom immune serum, 5 μL of pre-immune mouse serum, or 5 μL of unrelated antibody anti-BSA and used as IP control. Subsequently, 100 μL of protein G agarose slurry was added to the venom-antibody complex and incubated for 2 h at room temperature with gentle mixing. Then, 0.5 mL of IP buffer was added and centrifuged at 2500×*g* for 3 min, and the supernatant was discarded. The immune complex was neutralized with 50 μL of neutralization buffer (1 M Tris, pH 8.0), centrifuged again at 2500×*g* for 3 min, and 50 μL of 2X SDS-PAGE loading buffer was added for evaluation by SDS-PAGE electrophoresis in a 12% gel. The presence of the immunoprecipitated venom was determined by immunoblot using rabbit polyclonal anti-*L. laeta* venom serum (1:10,000 dilution) or anti-rLlPLD1 monoclonal antibody 7E4-D2 (1:50,000 dilution), then developed by incubating with goat anti-mouse IgG antibodies labeled with HRP (1:50,000) or goat anti-rabbit IgG antibody labeled with HRP (1:50,000) and by ECL.

### Hemolytic neutralization assay

The human erythrocyte hemolysis assay was performed as previously described [25]. Human erythrocytes were washed three times with veronal buffered saline (VBS^2+^ − pH 7.4; 10 mM sodium barbitone, 0.15 mM CaCl_2_, 0.5 mM MgCl_2_, and 145 mM NaCl) and resuspended at 2% in VBS^2+^. The cells were sensitized for 30 min at 37 °C with 25 μg/mL venom of *L. laeta* in 100 μL of VBS^2+^ in presence or absence of pooled serum from Group 1 and pooled serum from Group 2 at (1:1, 1:10 and 1:100 dilutions). Negative controls were incubated only with VBS^2+^. After incubation, the sensitized erythrocytes were washed three times with VBS^2+^ and were analyzed in a complement dependent hemolytic assay. Then, 100 μL of sensitized erythrocytes were mixed with 100 μL of normal human serum (NHS; 1:2 in VBS^2+^). The negative control was evaluated by incubating the erythrocytes with VBS^2+^ (without complement control) and total hemolysis control was incubated with H_2_O. After incubation for 1 h at 37 °C, the non-lysed cells were centrifuged at 440×*g* for 5 min, the supernatant was collected and measured at 414 nm. Results were expressed as a percentage of hemolysis. The assays were made in duplicate for a total of two independent experiments. The erythrocytes and normal serum were obtained from the same donor.

### Statistical analysis

Statistical analyses were performed using GraphPad Prism version 5.00 for Mac OS X (GraphPad Software Inc., La Jolla, CA, USA). Student t-test and One-Way ANOVA with Bonferroni Multiple Comparison post-hoc test was used to determine the statistical significance of differences among mean values. A statistical significance criterion significance level of *p* < 0.05 was used.

## Results

### Sera of people with no clinical history of loxoscelism recognize *L. laeta* venom

**Fig. 1:**
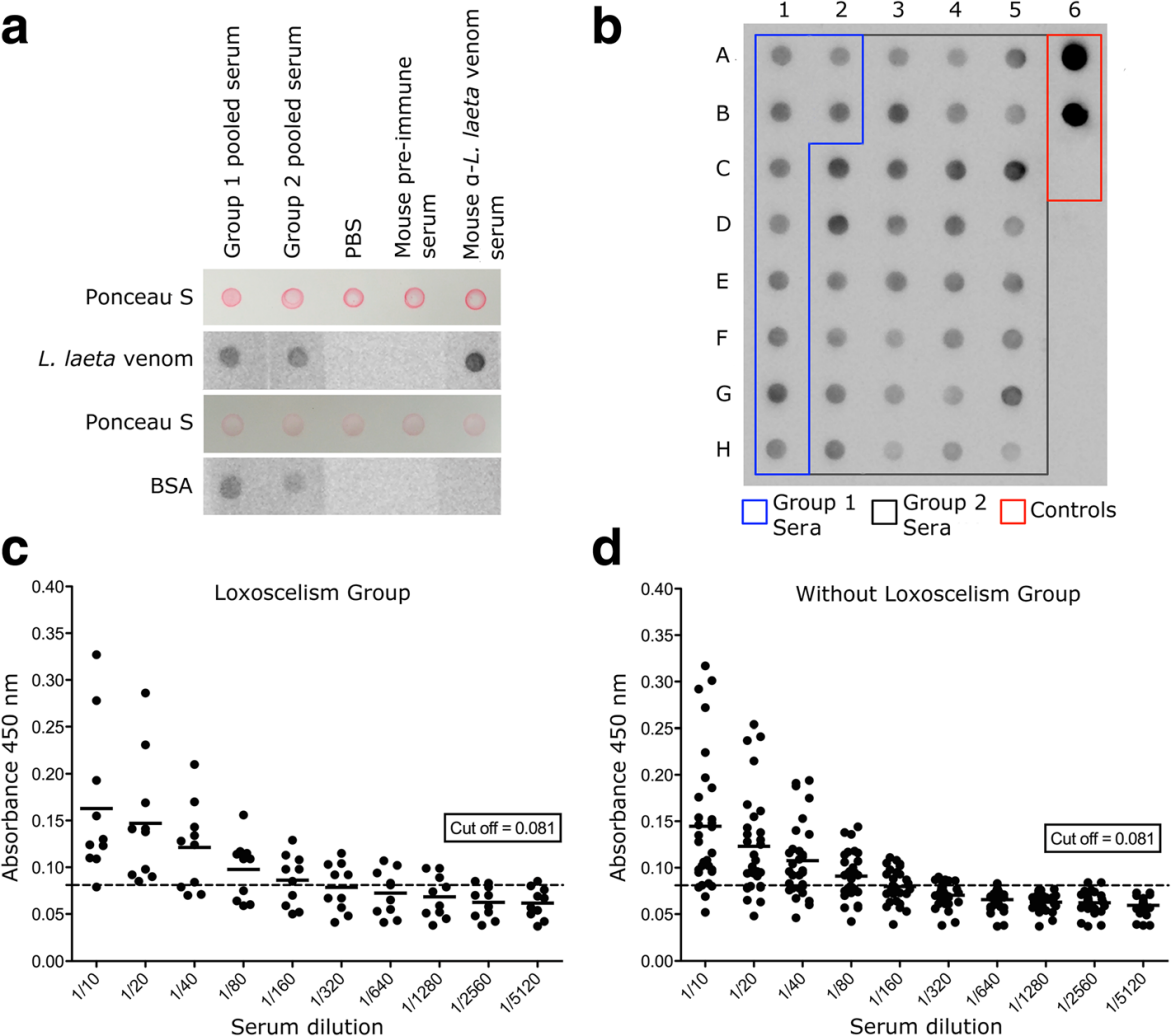
Detection of *Loxosceles* spider venom by sera from Group 1 and Group 2 by dot blot and ELISA. **a** Dot blot for detection of *L. laeta* venom incubated with serum pools from Group 1 and Group 2 (1:1000 dilution). **b** Representative dot blot of *L. laeta* venom incubated with individual serum from Group 1 (blue line, dots A1-B2) and Group 2 (black line, dots C2-H5); controls (red line): monoclonal antibody 7E4-D2 anti-rLlPLD1 (1:50,000 dilution) (dot A6), polyclonal mouse anti- *L. laeta* venom serum (1:10,000 dilution) (dot B6), pre-immune mouse serum (1:1000 dilution) (dot C6). **c** Indirect ELISA for the titration of Group 1 sera that recognize *L. laeta* venom. **d** Indirect ELISA for titration of sera from Group 2 that recognize *L. laeta* venom

In order to get a general view of immunoreactivity, the serum samples from individuals with loxoscelism (Group 1) and without loxoscelism (Group 2) were evaluated. Initially, the serum pools from Group 1 and Group 2 were used, and the detection of *L. laeta* venom by both groups was evaluated using dot blot. The pool of sera from the Group 1 was able to detect *L. laeta* venom. Meanwhile, the pool of sera from Group 2 was also able to recognize the venom (Fig. 1a). Incubation with PBS and pre-immune mouse serum did not show reactivity, whereas mouse anti-*L. laeta* venom serum showed a marked reaction.

In order to determine whether venom detection by the Group 2 pool of samples was due to the presence of individual serum that could present specific antibodies against *L. laeta* venom, we evaluated the detection of each individual’s serum using dot blot. All ten samples from patients with loxoscelism could detect *L. laeta* venom (Fig. 1b). In comparison, among the 30 samples from the group without loxoscelism, 18 of them showed detection levels similar to the sera from the loxoscelism group. In addition, five samples (dots C2, D2, B3, C3, C4, and C5) had higher detection levels than those observed in the loxoscelism group. In contrast, dots F3, A4, G4, H4, and H5 showed lower levels of detection (Fig. 1b; Additional file 1A). Strong detection was observed with mouse anti-*L. laeta* venom immune serum (dot A6) and monoclonal anti-rLlPLD1 (dot B6). Detection was not observed with pre-immune mouse serum (dot C6).

Additionally, titers of serums from both groups were evaluated by indirect ELISA. Briefly, each serum was diluted in the range of 1:10 to 1:5120. Absorbance values under the cut-off point (0.081) were considered to be non-specific or negative reactions. The detection of *L. laeta* venom for Group 1 serum sample titration media was 160 (*n* = 10) (Fig. 1c). Similarly, the media of titer for Group 2 sera was 80 (*n* = 30). Group 1 sera had absorbance values over the cut-off point for *L. laeta* venom detection at low dilutions (Fig. 1c). However, at 1:160 dilution, samples 1 (dot A1), 2 (dot A2), 8 (dot H1), and 10 (dot B2) presented lower values of the cut-off point. The majority of Group 2 sera had absorbance values over the cut-off (Fig. 1d). Samples 11 (dot G4), 14 (dot F3), 18 (dot A4), 19 (dot H5), and 26 (dot H4) had lower values of the cut-off point at 1:40 dilution. This is consistent with the results of the dot blot. However, the trend of the samples from the non-loxoscelism group (Group 2) remained above the cut-off point up to the titer 80. Additionally, avidity index for both pooled serums showed a high affinity with antibodies present in sera of groups 1 and 2 for detection of *L. laeta* venom (Additional file 2A).

In contrast, the total IgG concentration (mg/dL) of Group 1 and Group 2 sera was similar and within the reference range of the assay (Table 2). Total serum IgG levels of Group 1 samples ranged from 825 to 1622.5 mg/dL, and the total serum IgG levels of Group 2 samples ranged from 993.6 to 1902.9 mg/dL. The sample with lowest level of total IgG of group 1 sera was the sample 8 (dot H1), with a concentration of 825 mg/dL, while the sample with lowest level of total IgG from group 2 was the sample 14 (dot F3), with a concentration of 993.6 mg/dL. The latter correlates with data showed by dot blot and indirect ELISA, in which both samples had the lowest detection levels of *L. laeta* venom observed for both groups. Moreover, no differences in total IgG level were observed related to gender among samples.

**Table 2:** Human IgG levels in serum samples from patients with and without loxoscelism

Serum samples	IgG ^a^ (mg/dL)	Reference range (mg/dL)	*p*-value^#^
Loxoscelism group (n = 10)	1355 ± 117.1	710–1520	0.7445
Without loxoscelism group (n = 30)	1385 ± 36.14	710–1520	0.7445

^a^Values of media ± SEM. Human IgG concentration in serum were determined by radial immunodiffusion (RID). ^#^ t-test; *α =* 0.05

### Sera from individuals with no history of loxoscelism recognize phospholipase D family proteins from *L. laeta*

In order to confirm the above results, the *L. laeta* venom component that was specifically recognized by sera from groups 1 and 2 was evaluated. Each serum was assessed by immunoblot of *L. laeta* venom separated by electrophoresis, showing that all sera from Group 1 and Group 2 recognized a protein component between 25 and 35 kDa (Additional file 3). Sera of Group 2 that could recognize different bands of *Loxosceles* venom, compared to those in the range of 25–35 kDa proteins, were excluded on suspicion of previous contact with the *Loxosceles* venom.

**Fig. 2:**
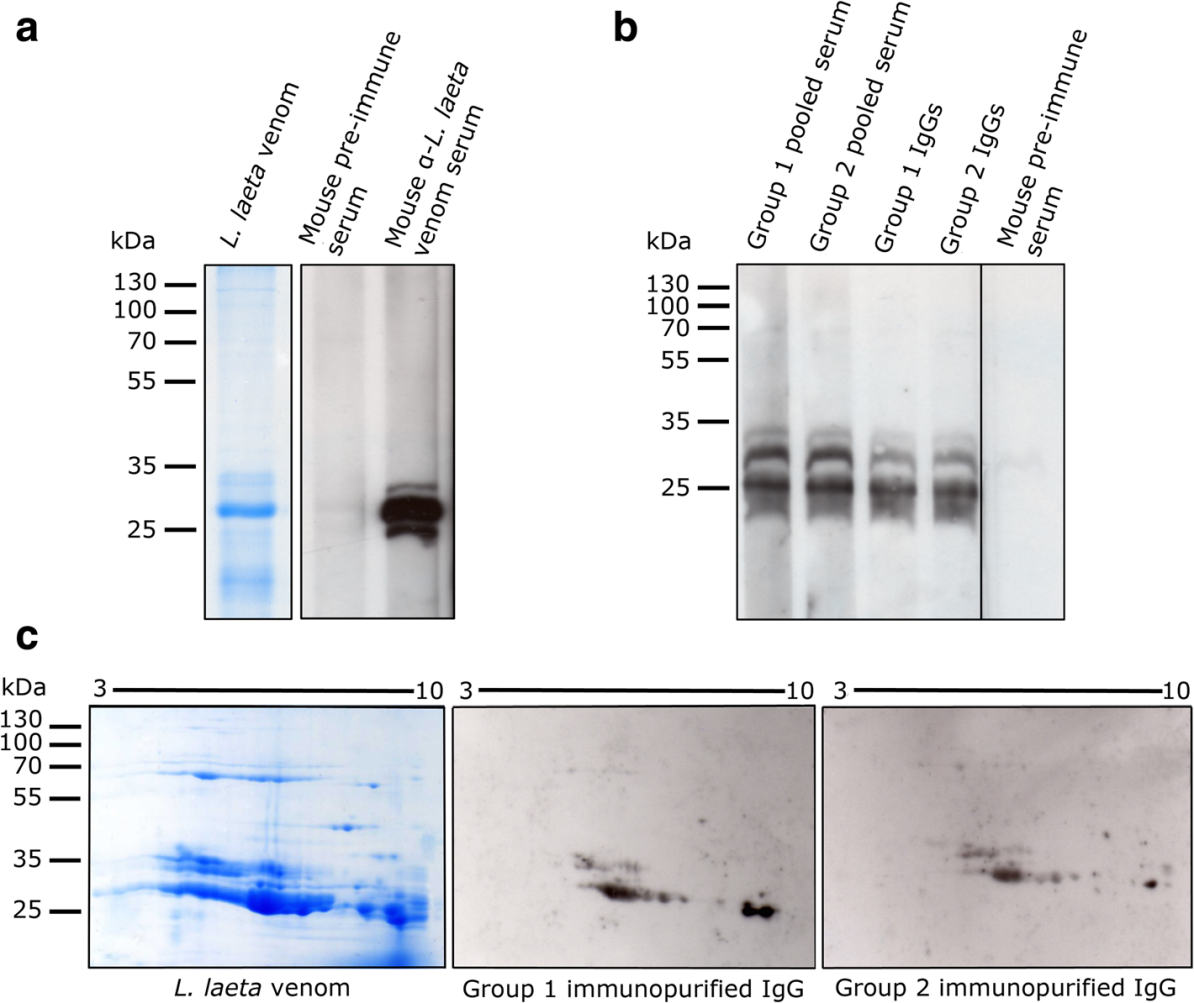
Immunoblot detection of *L. laeta* venom using pooled sera of Group 1 and Group 2. **a** Immunoblot detection of *L. laeta* venom with mouse anti-*L. laeta* venom immune serum. Lane 1: 12% SDS-PAGE of *L. laeta* venom stained with Coomassie brilliant blue. Lane 2: *L. laeta* venom immunoblot incubated with pre-immune mouse serum (1:1000 dilution). Lane 3: *L. laeta* venom immunoblot incubated with mouse *L. laeta* antivenom immune serum (1:10,000 dilution). **b** *L. laeta* venom immunoblot detected by pooled serum and purified IgGs of Group 1 or Group 2. Lanes 1 and 2: Serum pools for Group 1 and Group 2, respectively. Lanes 3 and 4: purified IgG antibodies (1 μg/mL) of Group 1 and Group 2 sera, respectively. Lane 5: pre-immune mouse serum. **c** Immunoblot of *L. laeta* venom separated by 2D electrophoresis

Additionally, considering that pooled sera from both groups recognized BSA in dot blot, and to discard reactions from antibodies other than anti-*L. laeta* venom in sera, we proceeded to purify IgG antibodies from the Group 1 and Group 2 serum pools and immunoselected against *L. laeta* venom. They were later evaluated by immunoblot for recognizing *L. laeta* venom separated by 1D and 2D electrophoresis (Fig. 2). As a detection control, mouse anti-*L. laeta* venom serum was used, noting that it recognizes a protein band pattern of *L. laeta* venom in the range of 25–35 kDa, while pre-immune mouse serum does not detect *L. laeta* venom (Fig. 2a), which was corroborated by densitometry analysis of bands (Additional file 1B).

Similar detection patterns of *L. laeta* venom were observed in Group 1 and Group 2 serum pools, as well as with purified IgG antibodies for both groups (Fig. 2b). By means of 2D venom electrophoresis, it was possible to see that the IgG antibodies of both study groups recognize a similar pattern of spots of *L. laeta* venom proteins, within the range of 25 and 35 kDa (Fig. 2c).

**Fig. 3:**
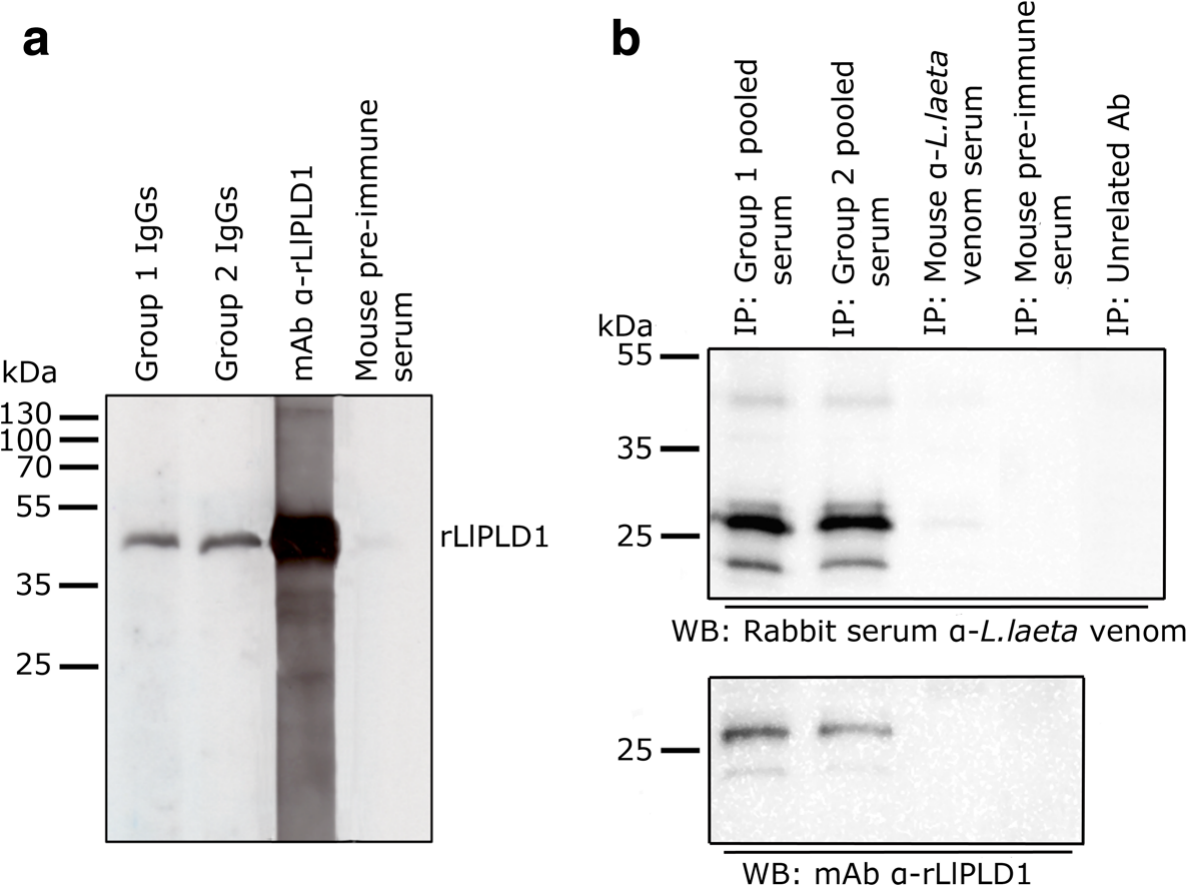
Immunoblot detection of recombinant LlPLD1 from *L. laeta* venom by heterophilic antibodies of groups 1 and 2. **a** Immunoblot detection of recombinant protein rLlPLD1 (5 μg) with purified IgGs (1 μg/mL) of sera from groups 1 and 2. Positive control comprised mAb anti-rLlPLD1-7E4-D2 (1:50,000 dilution) and negative control was pre-immune mouse serum (1:1000 dilution). **b** Immunoprecipitation (IP) of *L. laeta* venom with antibodies from groups 1 and 2 sera, and detection by (upper panel) immunoblot with rabbit polyclonal *L. laeta* antivenom serum (1:10,000 dilution) or (below panel) anti-rLlPLD1 monoclonal antibody 7E4-D2 (1:50,000 dilution). Mouse *L. laeta* antivenom serum, pre-immune mouse serum, and unrelated antibody anti-BSA were used as IP control

The protein components of *Loxosceles* venom between 25 and 35 kDa have been considered members of the phospholipase D family and are present in different *Loxosceles* species [30]. Therefore, we evaluated whether purified IgG antibodies from both groups could recognize the *L. laeta* phospholipase D1 protein (rLlPLD1), showing detection of the recombinant PLD with purified IgGs from both groups (Fig. 3a). In addition, *L. laeta* venom immunoprecipitation with pooled sera from groups 1 and 2 and subsequent immunoblot with a rabbit polyclonal anti-*L. laeta* venom serum (Fig. 3b, upper panel) or with monoclonal antibody anti-LlPLD1 (Fig. 3b, bottom panel) showed that PLD was the major protein immunoprecipitated from the venom. Moreover, since pooled sera from groups 1 and 2 could detect BSA in dot blot (Fig. 1a), we also carried out immunoprecipitation of *L. laeta* venom with an anti-BSA antibody as an unrelated antibody, which was not detected, corroborating the hypothesis that detection of *L. laeta* venom was a consequence of the presence of anti-PLDs antibodies in the serum samples of both groups. Additionally, mouse pre-immune serum was not able to immunoprecipitate *L. laeta* venom, and mouse polyclonal anti-*L. laeta* venom serum has only a low immunoprecipitation capacity of PLD of *L. laeta* venom. Based on these data, we can assume that the antibodies present in individuals with no clinical history of loxoscelism could correspond to heterophilic IgG-type antibodies, since there is no known previous exposure to the *Loxosceles* venom antigens in this group.

### Multispecificity of IgGs anti-PLD antibodies present in serum from individuals without history of loxoscelism

**Fig. 4:**
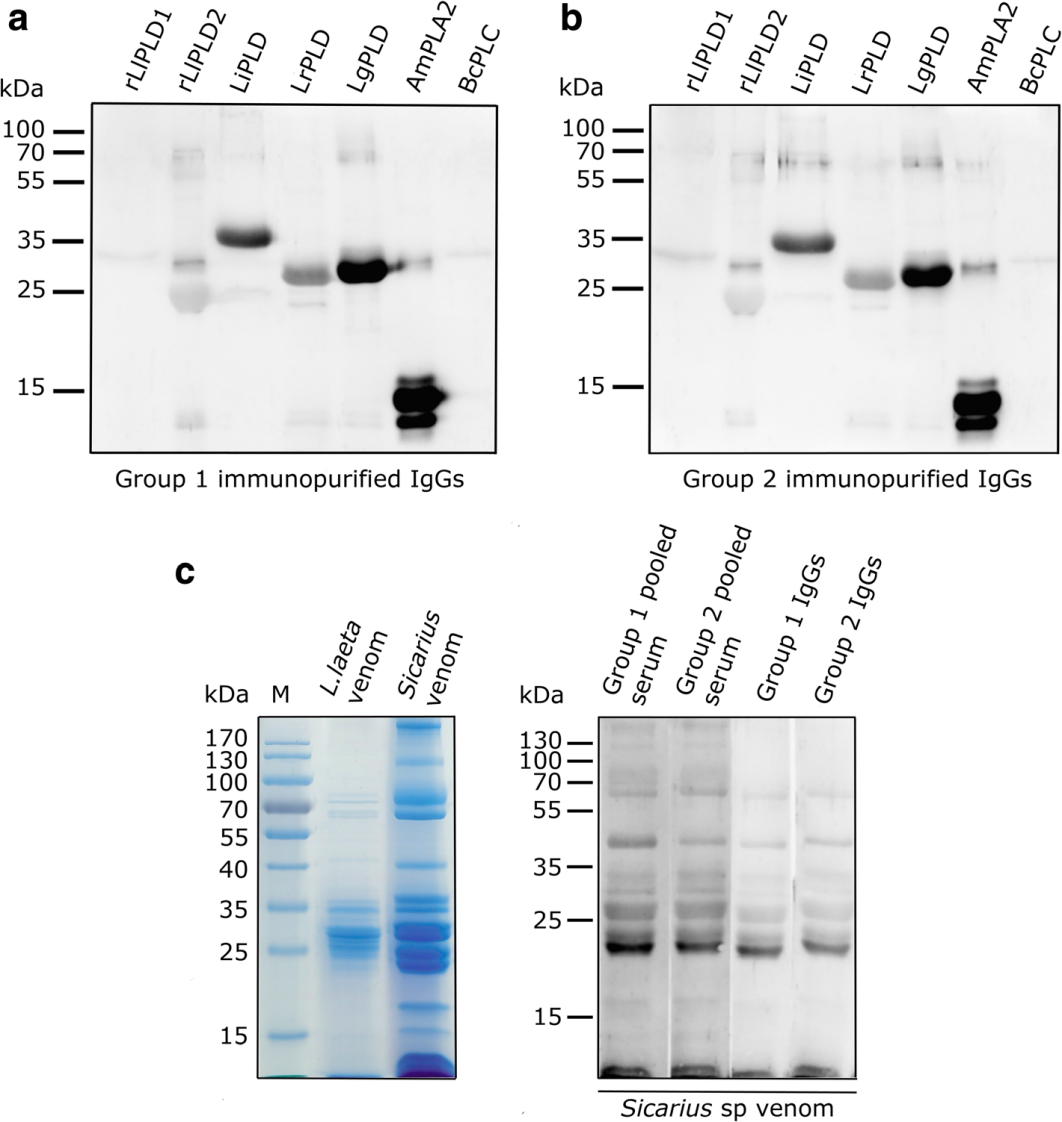
Heterophilic antibodies can detect PLD from other *Loxosceles* species and the venom from *Sicarius* spiders. A quantity of 5 μg of rLlPLD1, rLlPLD2, *L. intermedia* PLD (LiPLD), *L. reclusa* PLD (LrPLD), and *L. gaucho* PLD (LgPLD), together with 5 μg PLA_2_ of *Apis mellifera* venom (AmPLA_2_), and PLC of *Bacillus cereus* (BcPLC) were separated by SDS-PAGE in 12% gel and transferred to a nitrocellulose membrane. Then, each protein was detected by incubation with immunoselected IgGs from both groups at a concentration of 1 μg/mL, followed by incubated with goat anti-human HRP-IgG antibody (1:50,000 dilution), and developed with ECL. **a** IgG purified from Group 1 sera. **b** IgG purified from Group 2 sera. **c** Immunoblot from the venom of *Sicarius,* with serum pools and purified IgG antibodies of groups 1 and 2. (Left) SDS-PAGE in 12% gel of *L. laeta* venom and *Sicarius* venom stained with Coomassie blue. (Right) Immunoblot of *Sicarius* venom detected using serum pools from Group 1 and Group 2, and IgG antibodies purified from Group 1 and Group 2 sera

A second characteristic of heterophilic antibodies is it multispecificity. Consequently, we evaluated the multispecificity of these possibly heterophilic antibodies on the recognition of other phospholipase D isoforms from *L. intermedia*, *L. reclusa*, and *L. gaucho* through immunoblot. Thus, the purified and immunoselected IgG antibodies from both groups were able to detect different PLDs from other *Loxosceles* species, with a strongest detection against *L. intermedia* and *L. gaucho* PLDs (Fig. 4). This indicates a strong cross-immunoreaction of serum IgG antibodies from individuals with loxoscelism (Group 1) (Fig. 4a) and without loxoscelism (Group 2) (Fig. 4b) with the PLDs of these species. In addition, IgGs from both groups were able to strongly recognize phospholipase A_2_ (PLA_2_) from *Apis mellifera* venom, and weakly recognize phospholipase C (PLC) from *Bacillus cereus* (Fig. 4).

Then, we evaluated whether these IgG-type heterophilic antibodies could recognize venom of *Sicarius* spiders, a genus closely related to *Loxosceles*, which has paralogue PLD enzymes to those present in *Loxosceles* venom [31, 32]. The *L. laeta* venom and the *Sicarius* venom presented similar protein patterns (Fig. 4c), and both serum pools from groups 1 and 2, as well as the purified and immunoselected IgGs from both groups, were able to recognize the *Sicarius* venom (Fig. 4c).

Finally, we evaluated the neutralizing effect of these antibodies through a neutralization assay of *L. laeta* venom hemolytic activity, in which the non-neutralizing effect was observed for both pooled sera at different dilutions (Additional file 2B).

## Discussion

In the present study, we evaluated the presence of antibodies capable of detecting the venom of *Loxosceles* in serum samples from patients with (Group 1) and without (Group 2) loxoscelism. Surprisingly, it was possible to detect the presence of heterophilic antibodies capable of recognizing *L. laeta* venom in a group of control sera (people with no history of loxoscelism).

The antibodies found in people with no clinical history of loxoscelism not only were able to detect *L. laeta* venom in dot blot and ELISA tests, but also presented similar titers to serums from patients diagnosed with loxoscelism. In this latter group, the differences in observed detection level for individual serum could be due to different factors that influence in the severity of the clinical symptoms, and the developed immune response, as factors related to the spider, such as inter- and intra-species variations, spider developmental status, gender, and quantity of venom inoculated [7, 8, 33, 34]. In addition, there are patient factors, such as venom inoculation site, age and nutritional status [35].

Animal models inoculated with recombinant phospholipase D (the main immunogenic component of the venom), showed a significant increase of antibodies against venom in sera [25]. This antibody production is considered the basis for the development of neutralizing sera used as specific treatment [36], therefore, an increasing in IgG levels in patients with loxoscelism can be expected. However, the total IgG content in different analyzed sera showed no significant variations between both groups, and any differences due to sex. Therefore, exposure to *L. laeta* venom does not generate a significant increase in total IgG production in patients with loxoscelism. The latter is consistent with studies indicating that there is no relationship between the clinical picture of loxoscelism and IgG antibody levels in patient sera [23], and points towards the presence of natural or endogenous antibodies against the *Loxosceles* venom. In addition, our data showed that antibodies found in serum samples of people from both study groups had no neutralizing capacity. Consequently, they do not seem to influence the severity of the clinical picture of loxoscelism at hemolytic way. However, such observation requires further studies to determine the role of antibodies in the severity of the clinical picture, as for example, dermonecrosis.

From the three possible endogenous antibodies – heterophilic antibodies (HA), human anti-animal antibodies (HAAA), and autoantibodies – we believe that the antibodies present in sera from persons without loxoscelism do not appear to be autoantibodies, since the selection of individuals in this group included a criterion that would exclude people with a history of autoimmune diseases, especially rheumatoid factor. Also, the detection of *L. laeta* venom, both by dot blot and indirect ELISA, led us to believe that these antibodies do not correspond to human anti-animal antibodies, since these antibodies are known to be produced against animal immunoglobulins in people with history of immunotherapy. In addition, the serum samples from loxoscelism patient group used in our study were taken from patients who received no antivenom therapy, since Chilean guidelines for loxoscelism treatment does not suggest the use of antivenom therapy [2]. Thus, the detection of *Loxosceles* venom observed in this group was as a consequence of the presence of anti *L. laeta* venom antibodies produced by themselves and not the presence of antibodies from antivenom treatment, which could lead to production of HAAAs.

Certain future considerations and cautions should be taken about *Loxosceles* antivenom immunotherapy, since it involves the use of an anti-arachnid serum produced in horses [36], which could lead to the production of human anti-animal antibodies. In this regard, it has been documented the presence of anti-horse IgG antibodies in healthy volunteers without treatment with a horse antivenom used for the treatment of snakebites [37]. The presence and specificity of IgG antibodies in patients with loxoscelism undergoing serotherapy has been previously studied [23], showing that only results from four patients out of twenty that underwent serotherapy were able to detect the *L. gaucho* venom protein component of ~ 35 kDa by immunoblot. The authors indicate that the low number of patients able to recognize the venom was due to an inhibitory effect that sequesters the circulating immunogenic material. The authors also evaluated the sera through ELISA, in which the highest recognition titer of the venom was 1:640 and the lowest was 1:80 [23]. In our study, the mean titer for loxoscelism sera was 1:320, while the sera of patients without loxoscelism was 1:160, which is double the lowest titer reported by Barbaro et al. [23] for *L. gaucho* venom detection among loxoscelism patients. This indicates that the antibodies present in this group were possibly produced against an antigen similar to one of the components of the venom of *Loxosceles*, which present cross-reactivity immunodetection.

The third class of endogenous antibodies are the heterophilic ones, which are produced without exposure to a specific immunogen, so they can be considered as naturally occurring [21]. These antibodies are characterized by their multispecificity, being multireactive against heterogeneous or poorly defined antigens, and generally are often presumed to be low affinity antibodies, but this rule have exceptions [22]. Thus, the avidity index of antibodies in sera of both groups showed high affinity and led us to believe that these antibodies were produced early by an antigen with similar epitopes as *Loxosceles* PLDs. Despite having a high affinity for *L. laeta* venom, we think that antibodies found in sera from people without loxoscelism are heterophilic IgG antibodies, due to the unknown exposure to the antigen, which is considered a major criterion to consider an antibody as heterophile [20]. Therefore, we evaluated which *Loxosceles* venom components were detected by these possible heterophilic antibodies.

In our study, pooled sera from Group 1 (loxoscelism) and Group 2 (without loxoscelism), as well as IgG antibodies purified from both groups and immunoselected against *L. laeta* venom, could detect *L. laeta* venom proteins between 25 and 35 kDa, both in 1D and 2D immunoblot. The latter technique detected different proteins in this range, presumable indicating the multispecificity of these antibodies. Among the different protein components of the *Loxosceles* venom, the family of phospholipase D proteins (PLD) are capable of producing dermonecrosis, neutrophil activation, complement dependent red blood cell hemolysis, platelet aggregation, blood vessel permeability changes, kidney cytotoxicity, and recently it was demonstrated its role in monocyte recruitment [38, 39]. PLD molecular mass varies between 30 kDa and 35 kDa, and different isoforms of *Loxosceles* PLD have been documented for the different *Loxosceles* species [30, 40]. Our data showed that these IgG class antibodies detected the *L. laeta* recombinant protein phospholipase D1 (rLlPLD1), and other PLD isoforms of *L. intermedia*, *L. gaucho*, and *L. reclusa* indicating an important cross-immunoreactivity of antibodies present in the sera of individuals without loxoscelism, as well as that the specificity of the reaction was due to the presence of IgG antibodies capable of recognizing *Loxosceles* PLDs.

Likewise, this multispecificity was corroborated by cross-detection with other types of phospholipases, such as *Apis mellifera* phospholipase A_2_ (PLA_2_) and *Bacillus cereus* phospholipase C (PLC), which could indicate a common pattern of antigenicity among different types of phospholipases. Together with this, the IgG heterophilic antibodies from Group 2 recognized the venom of *Sicarius* spiders, which presented a venom protein pattern similar to that of *L. laeta*. These spiders are a closely related genus to *Loxosceles*, both belonging to the Sicariidae family [31, 41]. Both spider genera share important characteristics, such as similar venom protein patterns in the size range corresponding to known sphingomyelinase D (SMase D or PLD) proteins (31–35 kDa) and presence of active [30, 32]. Also, *Sicarius ornatus* exhibits venom interspecies differences at the gender level and has been documented as having active PLDs with complement dependent hemolytic activity in human red blood cells and cytotoxic activity in keratinocytes [42], similar to those reported for *Loxosceles* spiders [8]. In addition to this, serum anti-PLD of *L. intermedia* recognizes the 33 kDa component of *Sicarius* venom, which is a molecular mass also associated with PLDs in *Loxosceles* venom [42].

A relevant characteristic to consider an antibody as heterophilic is its unknown exposure to a specific immunogen [21]. In this regard, the possible origin of these natural or heterophilic antibodies in individuals without contact with *Loxosceles* venom is still unclear. However, the interspecies similarity between amino acid sequences of *Loxosceles* PLDs and the similarity in the venom protein patterns between the genus *Loxosceles* and *Sicarius* suggest that contact with the venom proteins of spiders closely related with *Loxosceles* could produce these antibodies [31]. Similarly, it cannot be ruled out that other arachnid genus could present PLDs capable of stimulating the production of these heterophilic antibodies. In addition, the antibodies could be produced due to previous exposure to bacterial PLDs, since the PLDs of *Loxosceles* spiders and bacteria such as *Corynebacterium pseudotuberculosis* possess similar molecular mass (31–32 kDa), have roughly 30% of sequence identity and have phospholipase activity on lysophosphatidylcholine (LPC) [43, 44].

The presence of SMase D (PLDs) in different pathogenic organisms, such as arachnids (genera *Acanthoscurria* and *Stegodyphus*), acarus (genera *Dermatophagoides*, *Varroa*, *Psoroptes*, and *Tetranychus*), ticks (*Ixodes scapularis*), bacteria (genera *Burkholderia*, *Streptomyces*, and *Austwickia*), and fungi (genera *Aspergillus*, *Fusarium*, *Coccidioides*, and *Trichophyton*, among others) has been reported and they share the same tridimensional structure as *Loxosceles* PLDs [45]. This would indicate that such a broad diversity of organisms with similar PLDs would facilitate the contact and production of antibodies capable of reacting with *Loxosceles* venom. This hypothesis is strengthened by the results observed in the present study for the immunodetection of heterophilic IgGs to *Bacillus cereus* PLC. Additionally, the origin of these heterophilic antibodies may be the exposure to other phospholipases, such as PLA_2_ from bee venom (*Apis mellifera*), since it has been reported that IgG_4_ antibodies present cross-reactivity for secreted PLA_2_s from different species, such as *Bos taurus* (cattle), *Apis mellifera* (honey bee), *Daboia russelii* (Russell’s viper), and *Naja mossambica* (spitting cobra) in patients allergic to *A. mellifera* venom and in control subjects [46].

The results presented in this study corroborate the presence of heterophilic IgG-class antibodies in the sera of individuals without loxoscelism. Interference caused by endogenous antibodies in sandwich immunoassays can occur by binding to, bridging, or blocking binding sites in capture or detection antibodies [22]. However, in the particular case of serum samples with the presence of anti-PLD antibodies, these could interfere in venom detection in a different way by directly binding to PLDs from *Loxosceles* venom. This would limit the binding of these proteins to the capture antibodies of sandwich ELISA or competitive type ELISA assays, due to a potential sequestration effect of venom components, which may lead to false negatives and underestimation of the presence of *Loxosceles* venom in patients. For this reason, considerations must be taken when blood and serum samples are used. Indeed, differences in venom detection, based on sample origin, have been documented in rabbits, in which detection of *Loxosceles* venom in hair samples, aspiration, and skin biopsy was possible for up to 7 days post-inoculation, but was undetectable in serum [47]. Along with our data, this observation point towards the use of skin samples over serum as a sample source for immunodetection tests of *Loxosceles* venom.

## Conclusions

In conclusion, in the present study we demonstrated the presence of IgG-class heterophilic antibodies directed against PLDs of *Loxosceles* and *Sicarius* spiders, present in people without contact with *Loxosceles* spider venom. The presence of these antibodies in serum samples should be considered as a possible interference in immunoassays for the specific detection of *Loxosceles* spider venom in humans.

## Additional files

Additional file 1: Densitometry analysis for dot blot and Western blot shown in Figs. 1b, 2a and b. Intensity of dots and bands were realized using ImageJ program, verifying for non-saturation and subtracting background. (A) Values from dots of Fig. 1b were expressed as relative density percentage calculated for each dot and normalized against control dot intensity with anti-*L. laeta* venom antibodies. Values are means ± S.E.M (*n* = 3). In addition, values of Western blot from Fig. 2a and Fig. 2b were expressed as relative density calculated from area mean density of each band and (B) normalized against the control band with mouse anti-*L. laeta* venom serum, (C) or normalized against control band with pool of serums of Group 1. Significance was evaluated with an ANOVA one-way with Bonferroni post-hoc test; (ns) indicates not statistically significant, and *** indicates significant differences between dots and control with *p* < 0.05. (TIF 27016 kb)

Additional file 2: Avidity index of pooled serums from Group 1 and Group 2, and neutralizing capacity of serums against hemolytic activity of venom of *L. laeta*. (A) Comparison of avidity index of pooled serums of Group 1 and Group 2 (1:100 diluted), treated with 6 M urea by IgG avidity ELISA. (ns) indicates not statistically significant. (B) Human erythrocytes were sensitized for 1 h at 37 °C with 25 μg/mL of venom of *L. laeta* in the presence or absence of pooled sera of Group 1 or Group 2 at 1:1, 1:10 and 1:100 dilutions, and evaluated in a complement-dependent hemolysis assay. Negative control was incubated only with VBS and with not presence of complement serum (control without complement). Results were expressed as percentage of hemolysis. The assays were made in duplicate for a total of two independent experiments and results are expressed as mean ± SEM. Significance was evaluated with an ANOVA one-way with Bonferroni post-hoc test; (ns) indicates not statistically significant. (TIF 9838 kb)

Additional file 3: Detection of *L. laeta* venom by immunoblot using single serums of Group 1 and Group 2. (A – Right) SDS-PAGE in 12% gel of *L. laeta* venom stained with Coomassie brilliant blue. (A – Left) Immunoblot detection of *L. laeta* venom incubated with mouse *L. laeta* antivenom immune serum (1:10,000 dilution) (CP). Immunoblot incubated with pre-immune mouse serum (1:1000 dilution) (CN). (B) *L. laeta* venom immunoblot detected by single serum from loxoscelism group (Group 1). (C) *L. laeta* venom immunoblot detected by individual serum from without loxoscelism group (Group 2). (TIF 2986 kb)

## Abbreviations

2D: two-dimensional
AA: autoantibodies
AmPLA2: PLA2 of *Apis mellifera* venom
BcPLC: PLC of *Bacillus cereus*
CL: cutaneous loxoscelism
HA: heterophilic antibodies
HAAA: human anti-animal antibodies
IP: immunoprecipitation
LgPLD: *Loxosceles gaucho* PLD
LiPLD: *Loxosceles intermedia* PLD
LPC: lysophosphatidylcholine
LrPLD: *Loxosceles reclusa* PLD
PLA_2_: phospholipase A_2_
PLC: phospholipase C
PLD: phospholipases D
RID: radial immunodiffusion
rLlPLD1: recombinant phospholipase D isoform 1
TMB: tetramethylbenzidine
VBS: veronal buffered saline
VCL: viscero-cutaneous loxoscelism
