# Supplementary figures and images for: Heterophilic antibodies in sera from individuals without loxoscelism cross-react with phospholipase D from the venom of Loxosceles and Sicarius spiders
Source: J Venom Anim Toxins Incl Trop Dis. 2018 Jul 26;24:18. doi: 10.1186/s40409-018-0155-x (PMC6062995; doi:10.1186/s40409-018-0155-x)

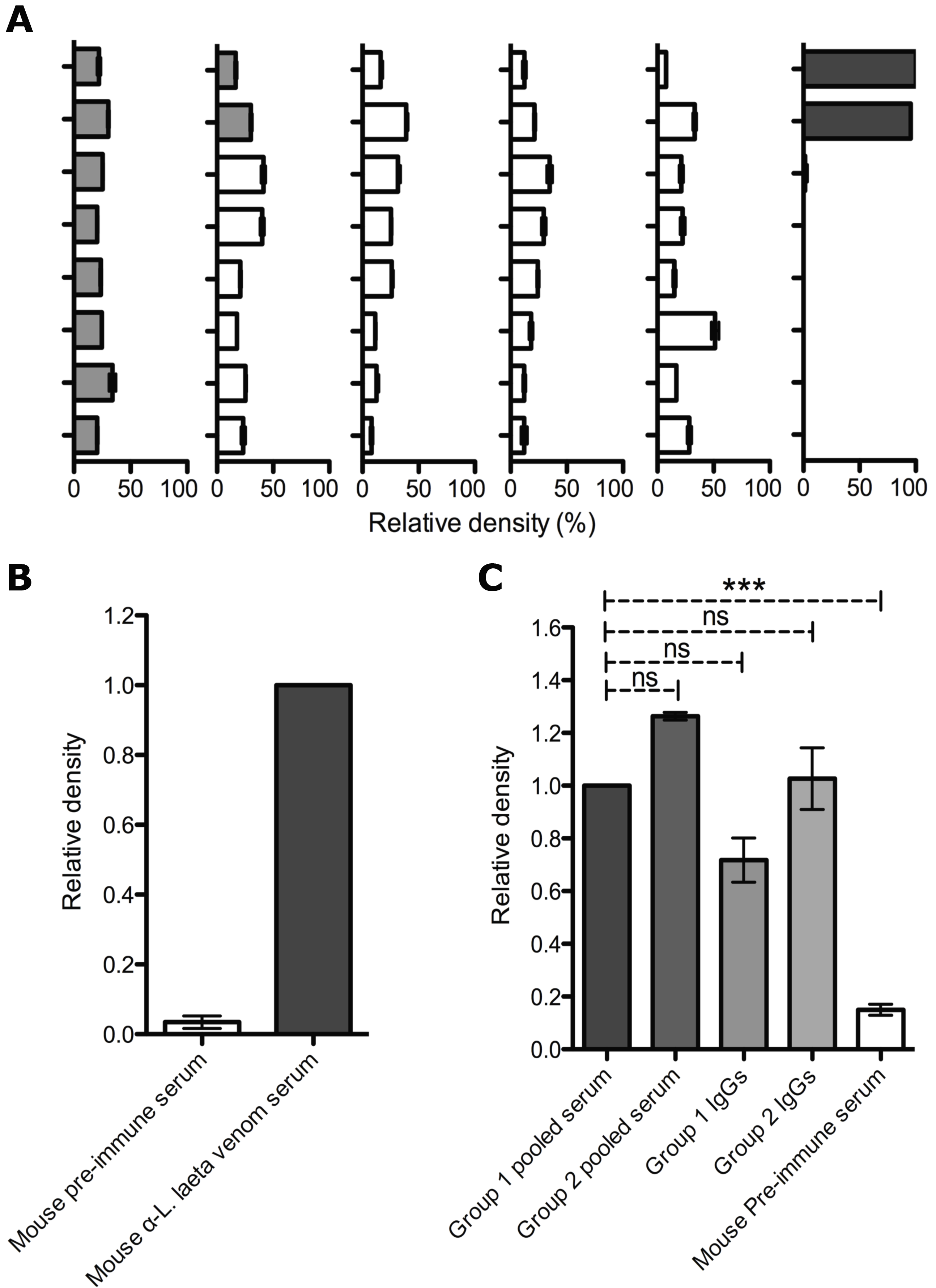

Supplement: Supplementary file 1 — Densitometry analysis for dot blot and Western blot shown in Figs. 1b, 2a and b. Intensity of dots and bands were realized using ImageJ program, verifying for non-saturation and subtracting background. (A) Values from dots of Fig. 1b were expressed as relative density percentage calculated for each dot and normalized against control dot intensity with anti-L. laeta venom antibodies. Values are means ± S.E.M (n = 3). In addition, values of Western blot from Fig. 2a and Fig. 2b were expressed as relative density calculated from area mean density of each band and (B) normalized against the control band with mouse anti-L. laeta venom serum, (C) or normalized against control band with pool of serums of Group 1. Significance was evaluated with an ANOVA one-way with Bonferroni post-hoc test; (ns) indicates not statistically significant, and *** indicates significant differences between dots and control with p < 0.05. (TIF 27016 kb) [file 40409_2018_155_MOESM1_ESM.tif]

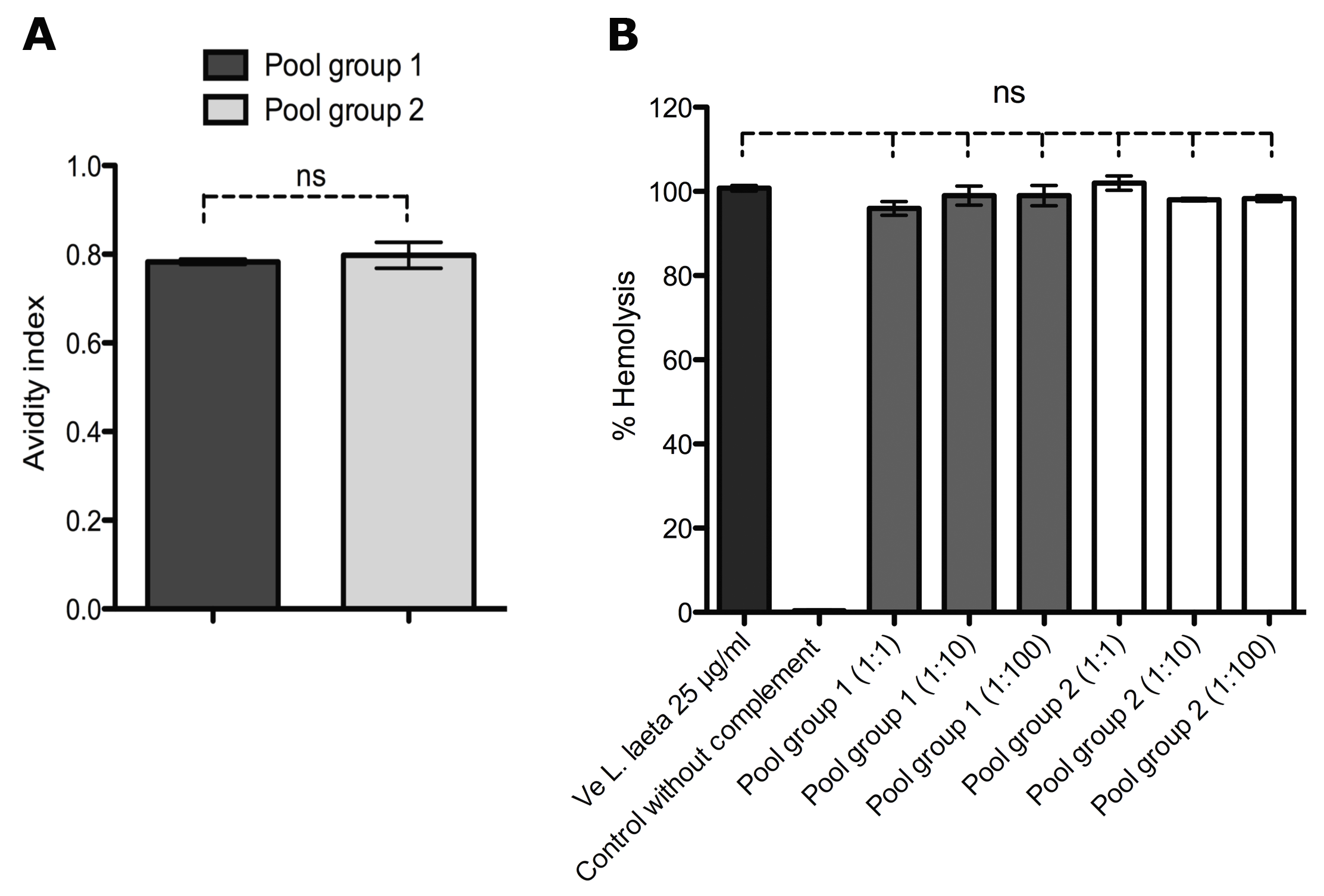

Supplement: Supplementary file 2 — Avidity index of pooled serums from Group 1 and Group 2, and neutralizing capacity of serums against hemolytic activity of venom of L. laeta. (A) Comparison of avidity index of pooled serums of Group 1 and Group 2 (1:100 diluted), treated with 6 M urea by IgG avidity ELISA. (ns) indicates not statistically significant. (B) Human erythrocytes were sensitized for 1 h at 37 °C with 25 μg/mL of venom of L. laeta in the presence or absence of pooled sera of Group 1 or Group 2 at 1:1, 1:10 and 1:100 dilutions, and evaluated in a complement-dependent hemolysis assay. Negative control was incubated only with VBS and with not presence of complement serum (control without complement). Results were expressed as percentage of hemolysis. The assays were made in duplicate for a total of two independent experiments and results are expressed as mean ± SEM. Significance was evaluated with an ANOVA one-way with Bonferroni post-hoc test; (ns) indicates not statistically significant. (TIF 9838 kb) [file 40409_2018_155_MOESM2_ESM.tif]

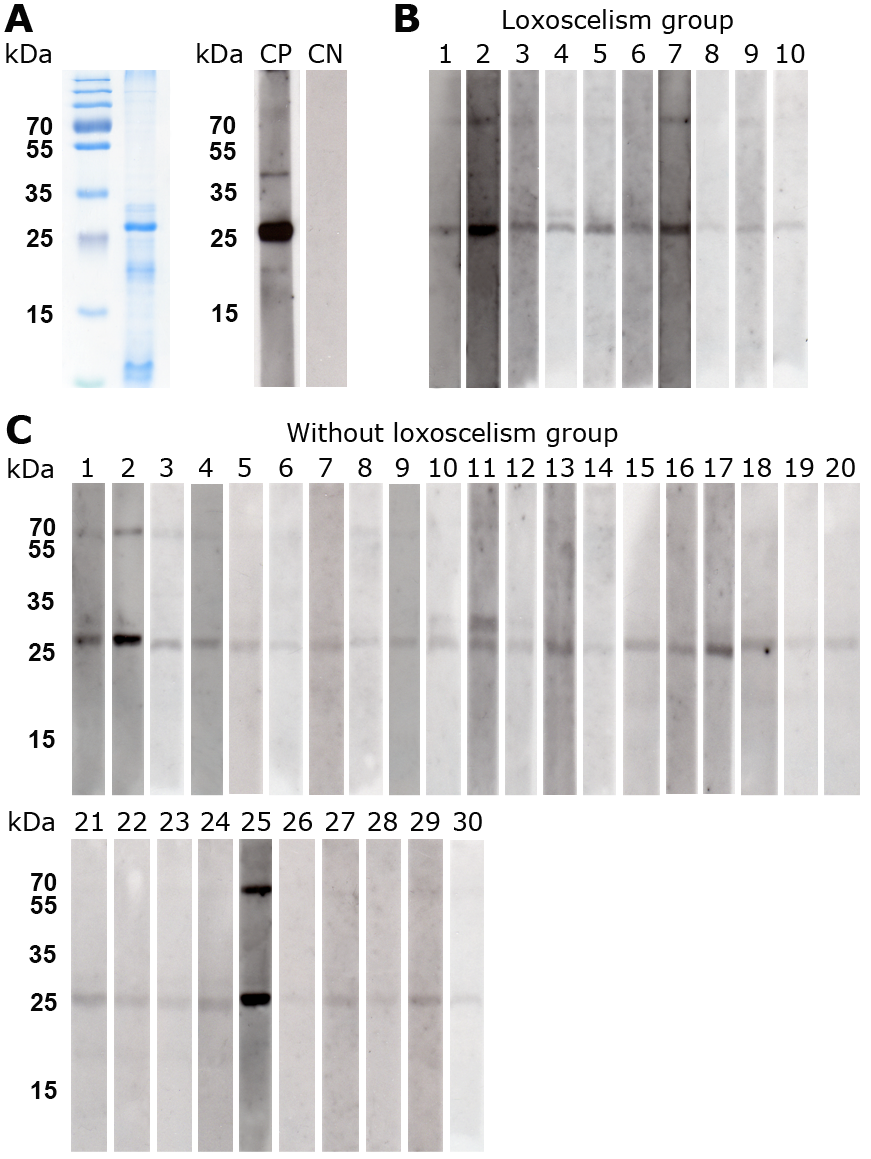

Supplement: Supplementary file 3 — Detection of L. laeta venom by immunoblot using single serums of Group 1 and Group 2. (A – Right) SDS-PAGE in 12% gel of L. laeta venom stained with Coomassie brilliant blue. (A – Left) Immunoblot detection of L. laeta venom incubated with mouse L. laeta antivenom immune serum (1:10,000 dilution) (CP). Immunoblot incubated with pre-immune mouse serum (1:1000 dilution) (CN). (B) L. laeta venom immunoblot detected by single serum from loxoscelism group (Group 1). (C) L. laeta venom immunoblot detected by individual serum from without loxoscelism group (Group 2). (TIF 2986 kb) [file 40409_2018_155_MOESM3_ESM.tif]
